# Thickness‐Dependent Thermal Conductivity and Phonon Mean Free Path Distribution in Single‐Crystalline Barium Titanate

**DOI:** 10.1002/advs.202301273

**Published:** 2023-04-24

**Authors:** Ankit Negi, Alejandro Rodriguez, Xuanyi Zhang, Andrew H. Comstock, Cong Yang, Dali Sun, Xiaoning Jiang, Divine Kumah, Ming Hu, Jun Liu

**Affiliations:** ^1^ Department of Mechanical and Aerospace Engineering North Carolina State University Raleigh NC 27695 USA; ^2^ Department of Mechanical Engineering University of South Carolina Columbia SC 29208 USA; ^3^ Department of Physics North Carolina State University Raleigh NC 27695 USA

**Keywords:** ferroelectric, mean free path, oxide perovskite, phonon, thermal conductivity

## Abstract

Nanosized perovskite ferroelectrics are widely employed in several electromechanical, photonics, and thermoelectric applications. Scaling of ferroelectric materials entails a severe reduction in the lattice (phonon) thermal conductivity, particularly at sub‐100 nm length scales. Such thermal conductivity reduction can be accurately predicted using the information of phonon mean free path (MFP) distribution. The current understanding of phonon MFP distribution in perovskite ferroelectrics is still inconclusive despite the critical thermal management implications. Here, high‐quality single‐crystalline barium titanate (BTO) thin films, a representative perovskite ferroelectric material, are grown at several thicknesses. Using experimental thermal conductivity measurements and first‐principles based modeling (including four‐phonon scattering), the phonon MFP distribution is determined in BTO. The simulation results agree with the measured thickness‐dependent thermal conductivity. The results show that the phonons with sub‐100 nm MFP dominate the thermal transport in BTO, and phonons with MFP exceeding 10 nm contribute ≈35% to the total thermal conductivity, in significant contrast to previously published experimental results. The experimentally validated phonon MFP distribution is consistent with the theoretical predictions of other complex crystals with strong anharmonicity. This work paves the way for thermal management in nanostructured and ferroelectric‐domain‐engineered systems for oxide perovskite‐based functional materials.

## Graphical abstract

High‐quality single‐crystalline barium titanate thin films are grown. The results show that the phonons with sub‐100 nm mean free path dominate the thermal transport in barium titanate, and phonons with mean free path exceeding 10 nm contribute ≈35% to the total thermal conductivity, in significant contrast to previously published experimental results.

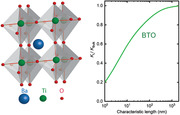

## 1 Introduction

Thermal conductivity (*k*) is an inherent property that dictates the heat flow in a material. In dielectric solids, thermal conductivity is predominantly due to phonons, normal modes of atomic vibrations.^[^ ^1^ ^]^The phonon dispersion relation (frequency and wavevector) and the phonon scattering, which characterize the propagation of phonons and the interactions among phonons, respectively, describe the behavior of phonons in a material. The phonon mean free path (MFP) distribution shows the contribution to thermal conductivity of phonons with different MFPs.^[^ ^2^ ^]^ With this information, we can tailor the thermal conductivity of materials using nano‐engineering approaches, or predict the reduced thermal conductivity in nanostructure because the contribution from phonons with MFP larger than the characteristic length of nanostructure is strongly suppressed.^[^ ^3^, ^4^, ^5^, ^6^ ^]^ The challenge, however, lies in evaluating the phonon MFP distribution using experimental or modelling approaches.^[^ ^7^ ^]^ Oxide perovskite (ABO_3_) based ferroelectrics are typical materials for such a study owing to the ubiquity of these materials as nanostructures and thin films. Further, the insights for these oxide perovskites serve as a standard to generalize the phonon MFP distribution of other complex crystalline materials with strong anharmonicity.

Oxide perovskite thin films, such as barium titanate (BaTiO_3_ or BTO), have been considered ideal candidates for several industrial applications.^[^ ^8^, ^9^, ^10^ ^]^ For instance, the strong ferroelectric behavior, that is, high room temperature polarization, observed in such epitaxially grown films shows their potential as lead‐free field‐effect transistors and in other ferroelectric/piezoelectric devices.^[^ ^11^, ^12^ ^]^ Adding to the impressive piezoelectric properties, their high dielectric constants and low dissipation further make them a suitable material for energy storage and conversion devices.^[^ ^13^, ^14^, ^15^ ^]^ More recently, such thin films and nanostructures have also been rendered functional in photonics due to their tunable optoelectronics properties coupled with physical hardness, stability, and variety of growth methods for large‐scale production.^[^ ^16^ ^]^ Studies have also explored other potential applications for these thin films including anti‐reflecting coating for resistive switching memories,^[^ ^17^ ^]^ superconductors,^[^ ^18^ ^]^ solar,^[^ ^19^ ^]^ photoelectrochemical water splitting,^[^ ^20^ ^]^ actuators for microelectromechanical systems,^[^ ^21^ ^]^ and in thermoelectric energy conversion.^[^ ^22^ ^]^

While the electrical, optical, and mechanical properties of BTO have been highlighted in studies, little attention has been paid to thermal properties despite the concern regarding thermal management in such devices.^[^ ^23^ ^]^As an example, thermal dissipation is critical for ensuring reliability in high‐energy‐density photonic network‐on‐chip devices.^[^ ^24^ ^]^ Variation in temperature alters the electro‐optical properties of the underlying material; thus, making thermal management vital in such cases. To ensure steady‐state operation of these high energy devices and mitigate thermal runaway, thermal conductivity of the material should be considered during device design. Of particular interest is the thermal conductivity of sub‐100 nm thin films or nanostructures, which is the typical feature size in several systems. The MFP of heat carriers (phonons) becomes comparable at the sub‐100 nm sizes, which can lead to significant reduction in thermal conductivity due to boundary scattering. Thus, knowledge of phonon MFP distribution is important for appropriate thermal management for such systems.

One way to construct the phonon MFP distribution is to measure thermal conductivity at several characteristic lengths. The characteristic length can be the film thickness for single crystalline films,^[^ ^25^ ^]^ feature size of nanostructures,^[^ ^26^ ^]^ and grain size for polycrystalline thin films.^[^ ^27^ ^]^ Experimentally, particularly for oxide perovskites, majority of the thin films reported in literature are polycrystalline because the growth of high‐quality perovskite thin films is highly susceptible to substrate, buffer layers, miscut, and chemical termination.^[^ ^28^ ^]^ The scantiness in experimental data makes it challenging to extract the phonon MFP distribution. For BTO, experimental thermal conductivity measurements are not only limited but also show inconsistent values. For bulk crystals, thermal conductivity values of 5.7^[^ ^29^ ^]^ and 3.6 W m^−1^ K^−1 [^ ^30^ ^]^ have been obtained for single crystals while 2.7^[^ ^31^ ^]^ and 10.2 W m^−1^ K^−1[^ ^32^ ^]^ have been reported for ceramics. For thin films, earlier measurements using the 3*ω* method show near bulk thermal conductivity for 100 nm polycrystalline BTO film with 150 nm average grain size (4.8 W m^−1^ K^−1^ for 100 nm film thickness compared to 5.15 W m^−1^ K^−1^ for 1 µm film thickness).^[^ ^33^ ^]^ It indicates that the average phonon MFP in BTO is much lower than 150 nm. However, recent measurements using the time‐domain thermoreflectance (TDTR) method for nanograined BTO films suggest a much higher average phonon MFP, showing more than 50% thermal conductivity reduction for an epitaxial 175 nm film thickness compared to bulk.^[^ ^34^ ^]^ Subsequent measurements using TDTR also show a similar high average phonon MFP.^[^ ^35^, ^36^ ^]^ Therefore, this information about the phonon MFP distribution in BTO is not conclusive in the literature due to the lack of consistency in the quality of grown films and discrepancy in the measured data. Moreover, simulations remain challenging due to either the inaccuracy of the force field in molecular dynamics or excluding the effects of higher‐order anharmonicity in the first‐principles based modeling.

Addressing such discrepancies in the phonon MFP distribution is vital not only for BTO but also for the study of other oxide perovskites. For instance, in a recent study,^[^ ^37^ ^]^ the thermal conductivity of La_0.5_Sr_0.5_CoO*_x_* was assumed to be constant in a thickness range of 8–16 nm. This is justified by the near linear MFP distribution in the thickness range calculated using harmonic dispersion and empirical scattering rates. Such approximations severely overestimate the average MFP, which challenges the feasibility of underlying assumption in the study. This highlights the need for the accurate prediction and measurement of phonon MFP distribution. Another example is found in strontium titanate (SrTiO_3_ or STO). Foley et al. measured the thermal conductivity of several polycrystalline STO thin films with grain sizes ranging from ≈30–90 nm. They observed that the thermal conductivity contribution of the ≈100 nm grain size was ≈50–60%.^[^ ^27^ ^]^ Similar results were also obtained for undoped and La: doped STO ceramics.^[^ ^38^, ^39^ ^]^ Theoretically, Feng et al. used a combined first‐principles molecular dynamics and anharmonic lattice dynamics approach to construct the phonon MFP distribution for STO.^[^ ^40^ ^]^ Despite accounting for only three‐phonon scatterings, their results indicate that a major portion of heat (≈80%) was carried by phonons with MFP less than 100 nm, contrary to the experimental results.

To address these discrepancies, we experimentally measured the through‐thickness thermal conductivity of high‐quality single‐crystalline [001]‐oriented BTO films in a thickness range of 14–56 nm and a bulk single crystal of 0.5 mm thickness at room temperature. We also calculated the phonon MFP distribution of BTO using ab initio molecular dynamics simulation and Boltzmann transport equation with the consideration of phonon scattering processes up to the fourth order. The calculated phonon MFP agrees well with our measured thickness‐dependent thermal conductivity data. We observed a dramatic rise in thermal conductivity around phonon MFP ≈ 10 nm, achieving near bulk thermal conductivity value for MFP ≈ 100 nm. Strong anharmonicity in the crystal structure of BTO made four‐phonon scattering important for reducing the average phonon MFP. The predicted MFP distribution was also in agreement with other strongly anharmonic complex crystal systems.

## 2 Results and Discussion

### 2.1 High‐Quality Single‐Crystalline BTO Thin Film

**Figure 1:**
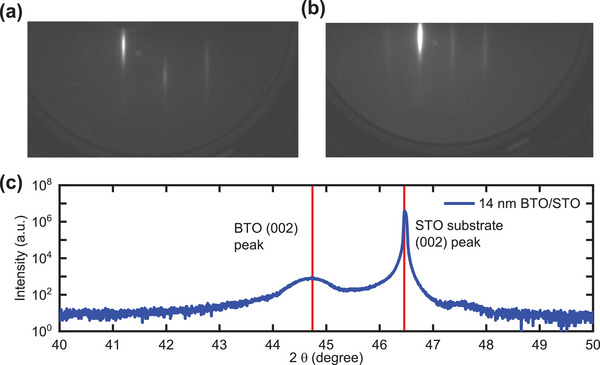
a,b) RHEED pattern of 32 nm‐thick BTO film grown on [001]‐oriented STO substrate by molecular beam epitaxy. The incident electron beam is along [100] for (a) and along[110] for (b). c) Representative X‐ray diffraction measurement data for a 14 nm‐thick BTO on [001]‐oriented STO around the (002) Bragg peaks of BTO film and STO substrate.

Single crystalline BTO films were grown on [001]‐oriented STO substrates by molecular beam epitaxy (MBE). Reflective high energy electron diffraction (RHEED) was used to monitor the growth of the layers. **Figure** **1**a,b shows the RHEED pattern at the end of growth of the 32 nm‐thick BTO film, where the sharp diffraction streaks indicate a high‐quality film. Figure 1c shows the X‐ray diffraction (XRD) peaks. The out‐of‐plane lattice constant of BTO film is calculated from the BTO (002) peak to be 0.4057 nm. The measured lattice parameter is larger than for the bulk BTO (*c* = 0.4038 nm),^[^ ^41^ ^]^ suggesting that the single phase tetragonal BTO film^[^ ^42^ ^]^ is grown under compressive strain with the polarization perpendicular to the film–substrate interface.

### 2.2 Determination of Thermal Conductivity for BTO

**Figure 2:**
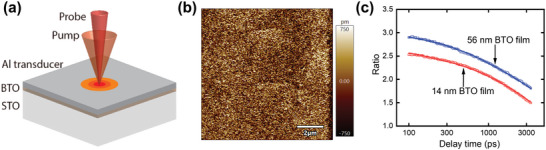
a) Schematic of thermal conductivity measurement of BTO thin films using TDTR. b) Surface morphology of the 14 nm BTO film scanned by AFM. The R.M.S. roughness of the film is ≈0.3 nm, indicating a smooth BTO growth over the STO substrate. c) TDTR normalized signal (ratio) for the 14 and 56 nm BTO thin films obtained using experiments (symbols), which are fitted with the heat diffusion model (solid lines). Ratio is calculated by dividing the in‐phase (*V*_in_) and out‐phase (*V*_out_) signals measured from the lock‐in amplifier, that is, ratio = − (*V*_in_/*V*_out_). With increasing film thickness, the thermal resistance increases, which results in a larger ratio.

The thermal conductivity of BTO at room temperature is determined using the time‐domain thermoreflectance method (TDTR).^[^ ^43^, ^44^ ^]^ **Figure** **2**a shows the schematics of this method, with more details in the Experimental Section. A thin layer of Al is deposited on top serving as both the heater and the reflectivity‐temperature transducer. The ultrafast laser beam is divided into pump beam and probe beam, where the pump beam heats the sample surface, and the probe beam measures the corresponding surface temperature rise via the surface reflectivity change. The time delay between the two beams allows us to track the transient temperature change upon heating, the rate of which depends on the thermal properties of the unknown layer (e.g., BTO layer). As TDTR is an optical method where surface reflectance is essential, the surface of the grown sample needs to be optically smooth with a typical requirement of R.M.S. roughness < 15 nm.^[^ ^45^, ^46^, ^47^ ^]^ Figure 2b shows the atomic force microscopy (AFM) scan of the surface on a representative sample and the surface roughness is below 1 nm. Such a smooth surface results from the high‐quality MBE growth of BTO on top of the STO substrate. Figure 2c shows two sets of typical TDTR experimental data where the normalized signals (also termed as “ratio”) are plotted as a function of the delay time between the two beams.

The cross‐plane (i.e., through‐thickness) thermal conductivities (*k*) of the single crystalline BTO films were obtained by fitting the experimental data from TDTR with the heat diffusion model. In theory, TDTR fitting involves the influence of several geometric, laser, and thermophysical parameters: the thickness of each layer, thermal conductivity, and heat capacity of each layer; interfacial thermal conductances between Al‐BTO (G_Al‐BTO_) and BTO‐STO (G_BTO‐STO_); and the laser spot size. The thickness of the Al transducer is determined using picosecond acoustics.^[^ ^48^ ^]^ The thickness of BTO is controlled and measured during growth. The thermal conductivity and heat capacity of Al are taken from literature, as in our previous work.^[^ ^49^ ^]^ The heat capacity of BTO is taken from literature.^[^ ^33^ ^]^ We measured the thermal conductivity of bare STO substrate (*k*_STO_ = 10.6 ± 0.4 W m^−1^ K^−1^), which agrees with the data reported previously.^[^ ^27^ ^]^ The heat capacity of STO is adopted from literature.^[^ ^50^ ^]^ Given the laser spot size in our experiment (5×, 1/e^2^ Gaussian beam radius ≈ 10.5 µm) is relatively large, the influence of this parameter on the signal is negligible. All these determined inputs leave us with three relevant undetermined thermophysical parameters in the fitting, the *k*_BTO_, G_Al‐BTO_, and G_BTO‐STO_.

**Figure 3:**
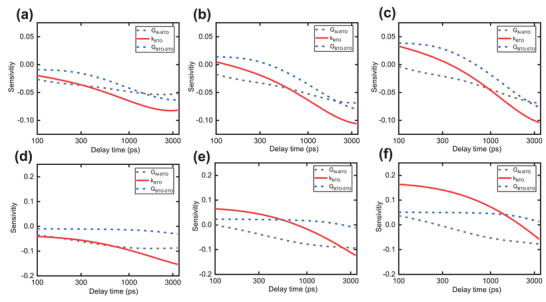
Sensitivity analysis for optimizing the measurement conditions. Sensitivity plots for 14 nm‐thick BTO thin film at a) 1 MHz, b) 3.85 MHz, and c) 7.2 MHz modulation frequencies. Sensitivity plots for 56 nm‐thick BTO thin film at d) 1 MHz, e) 3.85 MHz, and f) 7.2 MHz modulation frequencies.

To measure the *k*_BTO_ with a higher accuracy, we conducted a sensitivity analysis and then measured the samples with their selected modulation frequencies of pump beam. When varying the modulation frequency of pump beam, the thermal waves will penetrate different depths into the sample; thus, changing the relative sensitivities of these undetermined parameters. We calculated the sensitivity (*S*) of the three parameters at three representative modulation frequencies using Equation (1),

(1) $$S_{\alpha }=\frac{\partial }{\partial \alpha }\left(\frac{\ln R_{\alpha }}{\ln\alpha }\right)$$

where *α* is the undetermined thermophysical parameter and *R* is the TDTR signal (ratio). **Figure** **3** shows the sensitivity analysis results. For the thinnest sample (14 nm‐thick), at the low modulation frequencies, the signal is more sensitive to *k*_BTO_. However, the sensitivities of the interfaces (i.e., G_BTO‐STO_ and G_Al‐BTO_) are also comparable to that of *k*_BTO_. Extracting thermal conductivity of the BTO film, without any influence of interfaces, is thus challenging even with a multifrequency‐fitting approach (used in our previous work^[^ ^25^, ^43^ ^]^). At low modulation frequencies, other parameters including the specific heat of BTO, STO, and Al transducer; thermal conductivity of STO; and G_BTO‐STO_ also become more sensitive. Thus, the total uncertainty (more details in the next paragraphs) in determining *k*_BTO_ increases as well. In that regard, we chose a modulation frequency of 3.85 MHz for thinner samples (BTO film thickness < 40 nm) to balance the trade‐off between the uncertainty due to *k*_BTO_ sensitivity and uncertainties in other parameters. As the BTO film thickness increases, a relatively higher modulation frequency is preferred because of a smaller thermal penetration depth that leads to a predominantly higher sensitivity of the signal to *k*_BTO_. Thus, we selected a modulation frequency of 7.2 MHz for thicker samples (BTO film thickness > 40 nm).

In our case, as BTO is atomically grown on the STO substrate and the surface smoothness is ensured using RHEED, the interface can be assumed to be atomically smooth. Thus, we use the diffusive mismatch model (DMM) to calculate the interfacial thermal conductance between the BTO and STO interface at room temperature.^[^ ^51^ ^]^ We have demonstrated in our previous work that the DMM predicted interfacial thermal conductance is more accurate using first‐principles predicted full phonon dispersion relations compared with other approaches (e.g., using the Debye approximations). The second‐order force constants and dispersion relations for BTO and STO were extracted from the first‐principles method (with details in the Experimental Section). The calculated interfacial thermal conductance at room temperature for the BTO‐STO interface was 250 MW m^−2^ K^−1^. Using the DMM calculated G_BTO‐STO_ and given that both G_Al‐BTO_ and *k*_BTO_ are sensitive depending on the BTO thickness, we defined a parameter *σ* as in our previous work, to quantify the goodness of fit.^[^ ^52^ ^]^ *σ* is the summation of the standard deviation between the model and experimental data, at the modulation frequency (3.85 and 7.2 MHz in our case).

**Figure 4:**
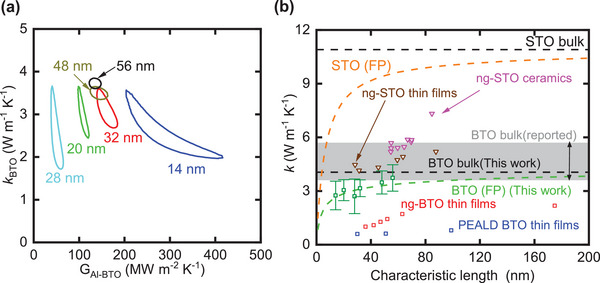
a) Contour plot of 2*σ* as a function of *k*_BTO_ and G_Al‐BTO_. For thinner films, the measured signal is less sensitive to the top interface, which translates to a larger uncertainty in G_Al‐BTO_. b) Thermal conductivity of BTO as a function of characteristic length. The characteristic length is equal to film thickness for this work (green squares) and PEALD BTO thin films (blue squares),^[^ ^36^ ^]^ and grain size for ng‐BTO thin films (red squares),^[^ ^34^ ^]^ ng‐STO thin films (brown triangles),^[^ ^27^ ^]^ and ng‐STO ceramics (pink triangles).^[^ ^38^ ^]^ The bulk value of BTO measured using a single crystal (black dashed line) is also shown. The reported values of BTO are also presented for reference (gray region).^[^ ^29^, ^30^, ^33^ ^]^ First‐principles (FP) predicted thermal conductivity as a function of characteristic length for STO (orange dashed line)^[^ ^40^ ^]^ and BTO (green dashed line). The predicted bulk value for STO is the top black dashed line. The error bars in the fitted BTO results are a summation of the 2*σ* uncertainty in *k*_BTO_ and standard TDTR error.

**Figure** **4**a shows the contour of 2*σ* (95% confidence interval) as a function of G_Al‐BTO_ and *k*_BTO_ for the BTO thin films. As shown in the sensitivity plots, the *k*_BTO_ is the most sensitive fitting parameter for thinner films at 3.85 MHz modulation, which leads to low uncertainty in *k*_BTO_. This uncertainty is a function of the signal‐to‐noise ratio and the quality of fit between the thermal model and experimental data. The total uncertainty in *k*_BTO,_ shown in Figure 4b, which also includes the systematic errors due to uncertainties in transducer film thickness, thermal properties of transducer film and substrate, spot size, and the BTO‐STO interfacial conductance. Even though we used the first‐principles‐enabled DMM to predict G_BTO‐STO_, we have considered a 20% input uncertainty for this parameter when computing the total uncertainty.

For the interfacial thermal conductance between Al and BTO, as the BTO film thickness increases, sensitivity plots show an increased dependence on G_Al‐BTO_, which translates to a lower uncertainty in the parameter. The range of our measured G_Al‐BTO_ lies within ≈50–250 MW m^−2^ K^−1^ for films with a thickness above 20 nm, which is reasonable for the interfacial thermal conductance for an evaporated metal–dielectric contact.^[^ ^53^, ^54^ ^]^ The high G_Al‐BTO_ for the 14 nm BTO can be related to the growth of BTO, Al transducer deposition, and the low sensitivity of this parameter. As the thermal evaporation of Aluminum on top of the BTO surface depends strongly on the evaporation condition and the quality of the sample surface, the thickness‐dependent interfacial thermal conductance between Al and BTO does not lead to any meaningful conclusion at such a small film thickness.

Figure 4b shows the fitted TDTR thermal conductivity of BTO thin films. We also measured the thermal conductivity of a bulk BTO single crystal (double sided polished procured from MTI Corporation) (*k*_BTO,bulk_ ≈ 4.05 ± 0.35 W^−1^ m^−1^ K) using TDTR. Substantial reduction in thermal conductivity was observed (≈30%) for the thinnest sample compared to bulk single crystal. Further, we observed a much steeper rise in thermal conductivity for the sub‐100 nm thin films, indicating a shorter MFP in comparison to BTO thin films reported in literature.

### 2.3 Modeling Phonon Transport in BTO

To understand the phonon transport and resolve the phonon MFP distribution in BTO, we performed first‐principles based modeling, including using density functional theory to optimize the unit cell, performing ab initio molecular dynamics simulations to obtain snapshots of atomic configurations and Boltzmann transport equation to calculate phonon transport properties (including phonon scattering rates) and thermal conductivity.

**Figure 5:**
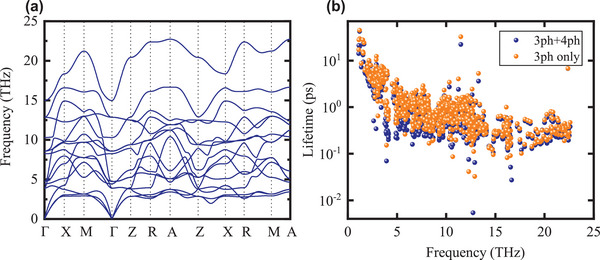
Phonon transport properties calculation results of BTO. a) Phonon dispersion relations. b) Phonon lifetime plotted in the log scale as a function of phonon frequency. The blue dots are phonon lifetimes considering both the three‐ and four‐phonon scattering processes in the calculation. The orange dots only consider the three‐phonon scattering processes.

**Figure** **5**a shows the calculated phonon dispersion curve for BTO. No negative frequencies appear in the dispersion curve, which indicates a proper relaxation of the cell. Figure 5b shows the phonon lifetime as a function of phonon frequency in BTO. The phonon lifetime is shorter when taking four‐phonon scattering processes into account. This observation is consistent with the studies in other materials, including Si,^[^ ^55^ ^]^ PbTe,^[^ ^56^ ^]^ and Bas,^[^ ^57^ ^]^ where the inclusion of four‐phonon scattering processes will decrease the overall phonon lifetime.

**Figure 6:**
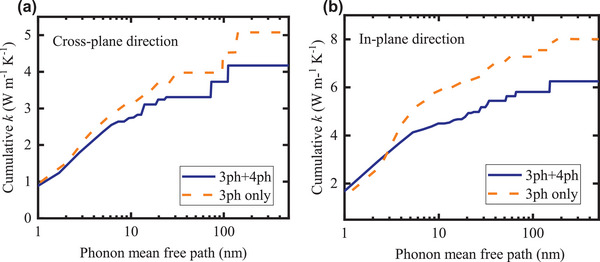
The cumulative thermal conductivity as a function of phonon mean free path in BTO at room temperature. a) Cross‐plane direction. b) In‐plane direction. The blue solid lines are the results considering both three‐phonon and four‐phonon scattering processes. The yellow dash lines are the results considering only the three‐phonon scattering processes.

**Figure** **6** shows the cumulative thermal conductivity as a function of phonon MFP in BTO at room temperature. For the cross‐plane direction (Figure 6a), the thermal conductivity reaches to 50% of bulk value at MFP ≈ 4 nm, 65% of bulk value at MFP ≈ 10 nm, 80% of bulk value at MFP ≈ 24 nm, and the bulk value (100%) at MFP ≈ 110 nm. Even though experimentally determining the in‐plane thermal conductivity of BTO thin films with a thickness of 14–56 nm is challenging, the first‐principles based calculations can provide insights in the in‐plane direction thermal transport. Figure 6b shows that the in‐plane thermal conductivity of BTO reaches to 50% of bulk value at MFP ≈ 2.5 nm, 80% of bulk value at MFP ≈ 27 nm, and the bulk value (100%) at MFP ≈ 150 nm. Moreover, the difference between with and without four‐phonon scattering processes indicates that considering the four‐phonon scattering process is necessary to achieve a more accurate cumulative thermal conductivity even at room temperature for BTO, a strongly‐anharmonic crystal.

### 2.4 Phonon MFP Distribution in BTO and Comparison With Other Complex Crystals

When comparing the modeling and measurement results in the cross‐plane thermal conductivity of BTO, we used a suppression function in the cross‐plane direction to convert from phonon‐MFP‐dependent thermal conductivity to film‐thickness‐dependent thermal conductivity. The details are described in Modeling Thickness‐Dependent Thermal Conductivity in the Experimental Section, and the result is plotted in Figure 4b. The predicted thickness‐dependent thermal conductivity in BTO at room temperature matches well with our measurement results, which validates the phonon MFP distribution in BTO.

Previous TDTR measurements on the nanograined‐BTO (ng‐BTO) thin film show more than twofold reduction in thermal conductivity compared to bulk single crystal, even for the epitaxial 175 nm thin film.^[^ ^34^ ^]^ An even higher reduction in thermal conductivity is observed for the plasma‐enhanced atomic layer deposition (PEALD) grown BTO thin films.^[^ ^35^, ^36^ ^]^ Both measurements are not consistent with our first‐principles thermal conductivity predictions. Similar discrepancy between the experimental measurements and first‐principles predictions is also observed in STO, also presented in Figure 4b.

We believe the reason for observing such large discrepancies are twofold, first, the growth of epitaxial film of perovskite oxides on a lattice mismatched substrate (e.g., Si) incurs structural imperfections and presence of large amorphous regions in the film.^[^ ^58^, ^59^, ^60^ ^]^ The actual phonon MFP thus becomes much smaller than the film thickness. This is observed in recent work during the growth of BTO on a Si substrate where the low thermal conductivity is attributed to mixed amorphous and crystalline phases.^[^ ^35^, ^36^ ^]^ Second, the experimental discrepancy can also be accounted for by considering the uncertainties in accurately determining the thermal conductivity of BTO film using TDTR measurement. The measurement is not only sensitive to the thermal conductivity of BTO but also to the interfacial thermal conductances. Lumping interfacial thermal conductance values together results in an effective thermal conductivity, that does not reflect the inherent *k*_BTO_. Again, assuming inappropriate value for *k*_BTO‐STO_ can result in lower‐than‐expected *k*_BTO_ values.

**Figure 7:**
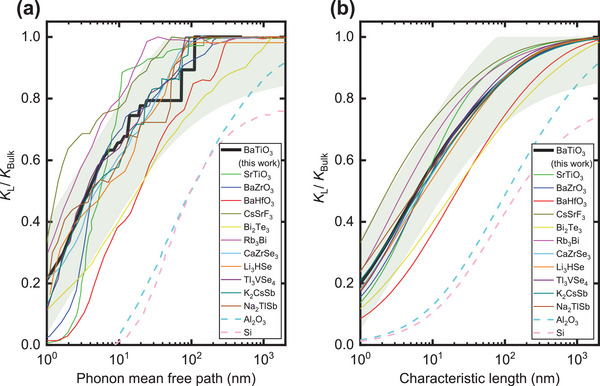
a) Comparison among the phonon MFP distribution curves in complex crystals with strong anharmonicity predicted at 300 K using first‐principles based calculations. b) Comparison among thermal conductivity variation as a function of characteristic length in complex crystals. The MFP is transformed to the characteristic length using the suppression function as described in Modeling Thickness‐Dependent Thermal Conductivity in the Experimental Section. The thermal conductivity can be tailored by controlling the characteristic length scale in the material, which can be the grain size, thin film thickness, or the limiting size of nanostructure. The data for complex crystals is taken from the following references: SrTiO_3_, BaZrO_3_,^[^ ^66^ ^]^ BaHfO_3_,^[^ ^67^ ^]^ CsSrF_3_,^[^ ^67^ ^]^ Bi_2_Te_3_,^[^ ^25^ ^]^ Rb_3_Bi,^[^ ^68^ ^]^ CaZrSe_3_,^[^ ^69^ ^]^ Li_3_HSe,^[^ ^70^ ^]^ Tl_3_VSe_4_,^[^ ^71^ ^]^ and K_2_CsSb.^[^ ^72^ ^]^ Two examples of simple crystals with relatively weak anharmonicity are also included here: Al_2_O_3_ (Sapphire)^[^ ^73^ ^]^ and Si.^[^ ^74^ ^]^ The green highlighted area indicates the 80% prediction interval for the phonon MFP distribution of BTO.

**Figure** **7**a shows a summary of the theoretically predicted normalized cumulative thermal conductivity ($k_{L}/k_{\mathrm{bulk}}$) as a function of phonon MFP in complex crystals, where the crystal has a complex chemical composition or atomic structure, and their strong anharmonicity has been demonstrated in the literature. Considering the differences in chemical composition and atomic structure in these complex crystals, we do not expect all the phonon MFP distribution curves will overlap. However, we surprisingly find that these curves share a similar shape and a few common features:

1) Phonons with MFPs longer than 1–10 nm still contribute significantly (20–60%) to the total thermal conductivity, which is contrary to the phonon MFP estimated from the simple kinetic theory based on phonon gas model.
2) Phonons with MFPs longer than 100 nm do not contribute substantially to the thermal conductivity, with <10% contribution in most complex crystals (only two exceptions in Bi_2_Te_3_ [≈25%] and BaHfO_3_ [≈18%]).
3) Phonons that contribute to the thermal conductivity have an MFP spanning in ≈two orders of magnitude. The slope on the plot is the highest around phonon MFP = 10 nm, which means the change in thermal conductivity is large when including or removing phonons with MFP around this range (5–20 nm). If these complex crystals are made into thin films, their thermal conductivity cannot be assumed to be the same in this range of film thickness when phonon‐boundary scattering becomes important.

These common features found in complex crystals with strong anharmonicity are in contrast with simple crystals with weak anharmonicity. For simple crystals with only one or two atoms in the cell and a simple cell structure, when the anharmonicity is weak, the phonon MFPs can be much higher (>>100 nm) and these phonons contribute significantly to the thermal conductivity. For example, in Si and sapphire (Al_2_O_3_), phonons with MFP larger than 100 nm contribute more than 50% to thermal conductivity and phonons with MFP smaller than 10 nm contribute negligibly. Phonons that contribute to the thermal conductivity have an MFP spanning in more than two orders of magnitude (e.g., 10–10^5^ nm in Si). This finding agrees with a recent work where Knoop et al.^[^ ^61^ ^]^ calculated the thermal conductivity and anharmonicity of crystalline solids using the fully anharmonic ab initio Green–Kubo first principles method. They found that the intrinsic strong anharmonic crystals will lead to a low thermal conductivity.

MFP distribution can also be applied to explore anomalous phonon transport, particularly in 1D and 2D materials.^[^ ^62^, ^63^, ^64^ ^]^ In the case of thin films, when the film thickness significantly exceeds the average phonon MFP, the thermal transport is typically diffusive. However, if the film thickness is smaller or comparable to the average phonon MFP, the thermal transport can be super‐diffusive, as demonstrated in previous studies.^[^ ^64^, ^65^ ^]^ In our case, the average phonon MFP is below 10 nm, which is smaller than most of the films measured in our study. Thus, we do not observe any significant effect of super‐diffusive phonon transport here.

The MFP‐dependent thermal conductivity is converted to characteristic‐length‐dependent thermal conductivity using the suppression function described in Modeling Thickness‐Dependent Thermal Conductivity in the Experimental Section. The characteristic length can be the average size of grains in polycrystalline materials, film thickness in thin films, and limiting or feature size of nanostructures. Figure 7b shows the suppressed thermal conductivity as a function of characteristic length, which serves as a guide for researchers to estimate the variation in thermal conductivity, a key piece of information in several applications. For instance, in thermoelectric materials, controlling the characteristic length to ≈10 nm can lead to a four to fivefold increase in thermoelectric figure of merit due to the substantial reduction in thermal conductivity, as shown in Figure 7b.^[^ ^25^, ^66^, ^68^ ^]^ Further, the length‐dependent thermal conductivity is also useful in developing thermal switches that exhibit dynamic modulation of thermal transport using ferroelectric domain wall engineering.^[^ ^1^ ^]^ The average size of ferroelectric domains can be selected based on the information provided in Figure 7b.

## 3 Conclusion

We measured the cross‐plane thermal conductivity of MBE‐grown single‐crystalline BTO thin films with a thickness of 14–56 nm at room temperature. The experimentally observed phonon mean free path distribution is corroborated by first‐principles based predictions. We find that, on the one hand, phonons with mean free paths larger than 100 nm contribute negligibly to thermal conductivity, contrary to the recent experimental observations. On the other hand, phonons with mean free paths larger than 10 nm contribute significantly (≈35%) to the total thermal conductivity, contrary to the kinetic theory based on the phonon gas model. The first‐principles based calculations reveal that the four‐phonon scattering process is non‐negligible for BTO, even at room temperature, which is likely due to the strong anharmonicity in the oxide perovskite structure. Considering the similarity in the phonon mean free path distribution with other strongly anharmonic crystals, this result will provide insights and a design guide on the thermal transport in ferroelectric materials and complex crystals. Our work paves the way for thermal management in nanostructured and ferroelectric‐domain‐engineered systems for a wide range of oxide perovskite based functional materials (e.g., ferroelectric materials and thermoelectric materials).

## 4 Experimental Section

### Materials Growth and Characterizations

Single crystalline BTO films were grown on [001]‐oriented STO substrates by molecular beam epitaxy (MBE). The samples were grown under 3.5 × 10^−6^ Torr oxygen plasma with the substrate temperature at 900 °C. Before the deposition, the STO substrates were annealed under 3.5 × 10^−6^ Torr oxygen plasma at 900 °C for 30 min to remove surface contaminants. High resolution X‐ray diffraction measurements were performed on a Rigaku SmartLab diffractometer. Before measuring the thermal conductivity, a thin layer of ≈80 nm Al (99.999%, Angstrom Engineering) was grown on top of the sample by thermal evaporation from a coated graphite source.

### TDTR Measurement of Thermal Conductivity

TDTR uses a mode‐locked Ti:sapphire laser that emits a train of femtosecond pulses at 80 MHz repetition rate. The laser is split into pump and probe beams, of which the former is modulated, and time delayed before being focused on the sample using an objective lens. The probe beam captures the periodic heating by probing the reflectivity change of transducer and at the pump modulation frequency for different delay times. The time decay of probed signal is used to determine thermal conductivity by fitting it with a multi‐layered heat conduction model. Further details regarding the experimental setup and model can be found in other studies.^[^ ^43^, ^49^ ^]^

### Modeling the Phonon Transport in BTO

First‐principles calculations were performed based on density functional theory (DFT) as implemented in the Vienna ab initio simulation package (VASP).^[^ ^75^ ^]^ The projector‐augmented wave (PAW) pseudopotentials were used to describe the interaction among atoms and the generalized gradient approximation (GGA) in the Perdew–Burke–Ernzerhof (PBE) form was chosen as the exchange‐correlation functional.^[^ ^76^, ^77^ ^]^ The kinetic energy cutoff of the plane‐wave function was set to 520 eV for BTO. The convergence criteria for optimization of BTO crystal structure, which has symmetry of P4mm and space group number of 99, were 10^−8^ eV and 10^−4^ eV Å^−1^ for the total energy and atomic forces, respectively. The optimization calculations fully allow the cell shape, cell volume, and atomic positions to change to reach the global minimum of potential energy surface of the BTO structure. The Brillouin zone was sampled using the Monkhorst‐Pack k‐mesh of 24 × 24 × 24, corresponding to the density of *k*‐points sampling of ≈0.16 Å^−1^ which was fine enough to guarantee high quality of DFT calculations.^[^ ^78^ ^]^ After structure optimization, a 4 × 4 × 4 supercell based on the five‐atom primitive cell with total number of atoms of 320, was generated. Ab initio molecular dynamics (AIMD) simulation for the supercell was then performed at 300 K by VASP. A total number of AIMD steps of 10 000 was run with timestep 1fs. The energy convergence criterion for supercell AIMD run was 10^−6^ eV with Brillouin zone sampled using Γ‐point. Consequently, among the total 10 000 snapshots of atomic configurations, 200 snapshots were randomly chosen, and the harmonic (2nd order) and anharmonic (3rd and 4th order) interatomic force constants (IFCs) were fitted by the temperature dependent effective potential (TDEP) method^[^ ^79^, ^80^ ^]^ with the higher‐order force constants truncated to 6 Å. With the 2nd order IFCs obtained, the phonon dispersions and phonon DOS were then calculated using PHONOPY.^[^ ^81^ ^]^ The lattice thermal conductivity of BTO structure was calculated using the ShengBTE package.^[^ ^82^ ^]^ Four‐phonon scattering was considered by using the 4th order IFCs obtained.

### Modeling Thickness‐Dependent Thermal Conductivity

When comparing the modeling and measurement results, a suppression function was used in the cross‐plane direction to convert from phonon‐MFP‐dependent thermal conductivity to film‐thickness‐dependent thermal conductivity. Here, how the suppression function was considered to calculate the film‐thickness‐dependent thermal conductivity, is briefly described. In the case of thermal conductivity in nanostructures *k*_nano_,

(2) $$k_{\mathrm{nano}}=\int_{0}^{\infty }K_{\lambda }Bd\lambda_{\mathrm{bulk}}$$

where *λ*_bulk_is the mean free path for the bulk crystal, *K*_*λ*_ is the mean free path spectrum for the bulk crystal, and *B* is the suppression function. Here

(3) $$B\left(Kn\right)=B\left(\frac{\lambda_{\mathrm{bulk}}}{L_{c}}\right)=\frac{\lambda_{\mathrm{nano}}}{\lambda_{\mathrm{bulk}}}$$

where *Kn* is the Knudsen number, *L*_c_is the characteristic length of the nanostructure, and *λ*_nano_ is the mean free path for the nanostructure. Here, both the*λ*_bulk_ and*K*_*λ*_ are obtained from the first‐principles calculations. For the cross‐plane thermal conductivity of a thin film with thickness *d*, the suppression function is adopted from Ref. [83].

(4) $$B_{\mathrm{film}}\left(Kn\right)=1+3Kn\left[E_{5}\left(Kn^{-1}\right)-0.25\right]$$

where, *E*_5_ is the 5th‐order exponential integral and*Kn* = *λ*_bulk_/*d*.

## Conflict of Interest

The authors declare no conflict of interest.

## Data Availability

The data that support the findings of this study are available from the corresponding author upon reasonable request.
